# Spatiotemporal auxin distribution in Arabidopsis tissues is regulated by anabolic and catabolic reactions under long-term ammonium stress

**DOI:** 10.1186/s12870-021-03385-9

**Published:** 2021-12-18

**Authors:** Kacper Dziewit, Aleš Pěnčík, Katarzyna Dobrzyńska, Ondřej Novák, Bożena Szal, Anna Podgórska

**Affiliations:** 1 Institute of Plant Bioenergetics, Faculty of Biology, University of Warsaw, I. Miecznikowa 01, 02-096 Warsaw, Poland; 2 Laboratory of Growth Regulators, Faculty of Science, Palacký University and Institute of Experimental Botany, The Czech Academy of Sciences, Šlechtitelů 27, CZ-78371 Olomouc, Czech Republic

**Keywords:** Ammonium nutrition, *Arabidopsis thaliana*, Auxin conjugation, Auxin degradation, Auxin synthesis, Root development

## Abstract

**Background:**

The plant hormone auxin is a major coordinator of plant growth and development in response to diverse environmental signals, including nutritional conditions. Sole ammonium (NH_4_^+^) nutrition is one of the unique growth-suppressing conditions for plants. Therefore, the quest to understand NH_4_^+^-mediated developmental defects led us to analyze auxin metabolism.

**Results:**

Indole-3-acetic acid (IAA), the most predominant natural auxin, accumulates in the leaves and roots of mature *Arabidopsis thaliana* plants grown on NH_4_^+^, but not in the root tips. We found changes at the expressional level in reactions leading to IAA biosynthesis and deactivation in different tissues. Finally, NH_4_^+^ nutrition would facilitate the formation of inactive oxidized IAA as the final product.

**Conclusions:**

NH_4_^+^-mediated accelerated auxin turnover rates implicate transient and local IAA peaks. A noticeable auxin pattern in tissues correlates with the developmental adaptations of the short and highly branched root system of NH_4_^+^-grown plants. Therefore, the spatiotemporal distribution of auxin might be a root-shaping signal specific to adjust to NH_4_^+^-stress conditions.

**Supplementary Information:**

The online version contains supplementary material available at 10.1186/s12870-021-03385-9.

## Background

Nitrogen acquisition significantly affects plant growth [1], yet it is not always the quantity of nitrogen that matters, but rather the form of nitrogen that is acquired by plants. Inorganic nitrogen resources commonly available for plants include nitrate (NO_3_^−^) or ammonium (NH_4_^+^) ions. However, when NH_4_^+^ is the sole source of nitrogen for plants, serious toxicity symptoms develop, leading to a condition known as the ammonium syndrome [2, 3]. At the plant level, this syndrome is mainly characterized by significant retardation in development, lower biomass, less seed establishment, and shorter primary roots, all adding up to lower plant yield, which is very unfavorable in the context of crop cultivation. Therefore, it is desirable to overcome this adverse status to achieve better plant production from higher NH_4_^+^ fertilization rates. Many hypotheses have been proposed to explain why NH_4_^+^ might have a cumulative toxic effect on plants, causing energy deficiency, limited cell wall expansion, carbohydrate shortage, pH disturbances, ion imbalances, oxidative stress, and other symptoms [4–7]. Despite years of research dedicated to understanding why growth arrest might be expected under NH_4_^+^ nutrition, the cause remains unclear.

The plant hormone auxin is one of the fundamental regulators of plant growth and development. It plays a key role in maintaining apical dominance and controlling plant tropism as well as in the development of leaves, roots, and floral organs [8–10]. Mostly auxin is known for its impact on root morphology because of its role in initiating lateral root development and apical root cell differentiation [11–13]. In contrast, the main sites of auxin synthesis occur in aboveground plant parts, such as stem apexes and developing leaf primordia [14]. Indole-3-acetic acid (IAA) is the major natural form of auxin in plant tissues, and it’s content is regulated by various developmental and environmental cues. The main pathway of de novo IAA synthesis is the tryptophan (Trp) pathway [15]. In its major route Trp is converted into indole-3-pyruvate (IPyA) by the Trp aminotransferase of Arabidopsis (TAA1) and Trp aminotransferase related (TAR1-2). Subsequently, IPyA is transformed into IAA by the YUCCA family of flavin monooxygenases (YUC1-11). Additionally IAA can be synthetized in an IPyA independent pathways containing for instance the intermediates indole-3-acetamide (IAM) and indole-3-acetonitrile (IAN) or tryptamine (TAM). Also, an entire Trp independent pathway is possible, branching of its precursor anthranilate (ANT) [16].

Only a short-lived fraction of the synthesized auxin pool remains in its free form in plants [17]. Auxins can be quickly incorporated into conjugates with various compounds such as amino acids, sugars, and peptides; depending on their partners, auxin conjugates are intended either for storage or degradation [18]. The formation of amide-linked IAA conjugates is catalyzed by Gretchen Hagen 3 (GH3) family proteins consisting of 19 enzymes in *Arabidopsis thaliana*. The combination of IAA with alanine (Ala) or leucine (Leu) is a storage form, while its combination with asparagine (Asp) or glutamate (Glu) is a precursor for degradation [17]. Furthermore, IAA and its oxidized form (oxIAA) can form conjugates with glucose (Glc) via a glycosylation reaction catalyzed by heterogeneous UDP-glycosyltransferase (UGT) superfamily enzymes [19]. The resulting conjugate, IAA-Glc, can be stored in tissues, while its oxidized form oxIAA-Glc is intended for degradation. Auxin can also be directly oxidized by dioxygenase of auxin oxidation (DAO1-2), and the resulting auxin catabolite is oxIAA [20]. Overall, IAA oxidation occurs very fast, so the remaining free IAA can be considered a signaling element [21]. Therefore, changes in auxin homeostasis are thought to underlie environmental stress communication that regulates plant growth.

Previously, it was speculated that NH_4_^+^-based developmental retardation of plants may be related to hormonal imbalances [5, 6]. Since then, auxins have been in the spotlight to affect NH_4_^+^-mediated growth phenotype. The research on auxins has focused primarily on root morphology because they have long been considered as the rooting hormone. A pronounced auxin response was found in roots of most plants subjected to NH_4_^+^ nutrition [22–26], but because these studies focused only on the primary root or young seedlings, the conclusions can only be tentative. However, to date, the reason for the observed changes in auxin pools in tissues of NH_4_^+^-exposed plants has not been fully elucidated.

To address the above question, we analyzed the pathways of auxin synthesis, conjugation, and oxidation. These major mechanisms can shed light on events that regulate the steady state of auxin in tissues of plants grown with NH_4_^+^ as the sole source of nitrogen. In this study, we examined auxin metabolism in the shoots and roots of mature Arabidopsis plants subjected to NH_4_^+^ nutrition. Our results help understand how balancing auxin pools *in planta* can be the basis of the NH_4_^+^ syndrome development.

## Results

### Ammonium cultivation caused reduced rosette development and a characteristic short and highly branched root phenotype

**Fig. 1:**
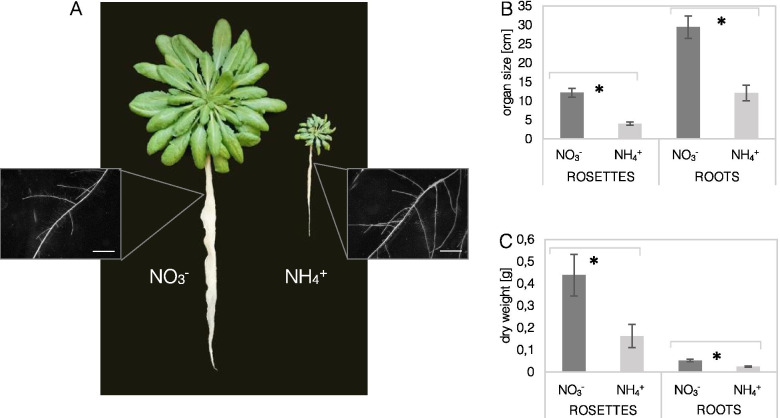
Phenotype of *Arabidopsis thaliana* (Col-0) after 8 weeks of hydroponic culture in a solution containing 5 mM NO_3_^−^ (control) or 5 mM NH_4_^+^ as the sole nitrogen source. **A** Visual comparison of plants and magnifications of roots under binocular (insert; scale bars = 1 mm). **B** Rosette diameter and primary root length. **C** Dry weight of leaves and roots. Means are given with their SD (*n* = 10). Asterisks represent statistically significant differences between NO_3_^−^ versus NH_4_^+^-derived tissues (*P* < 0.05)

Arabidopsis plants fed with NH_4_^+^ exhibited smaller rosettes and shorter roots, and also smaller rosette diameter and primary root length values, compared to the plants fed with NO_3_^−^ (Fig. 1A, B). However, the roots of the plants grown with NH_4_^+^ showed a highly branched architecture with many lateral roots (Fig. 1A; Supplemental Fig. 4). In addition, the leaves and roots of these plants showed more than twice lower dry weight in the presence of NH_4_^+^ (Fig. 1C).

### Staining of auxin reporters had a higher intensity in leaves and was heterogeneous in roots

**Fig. 2:**
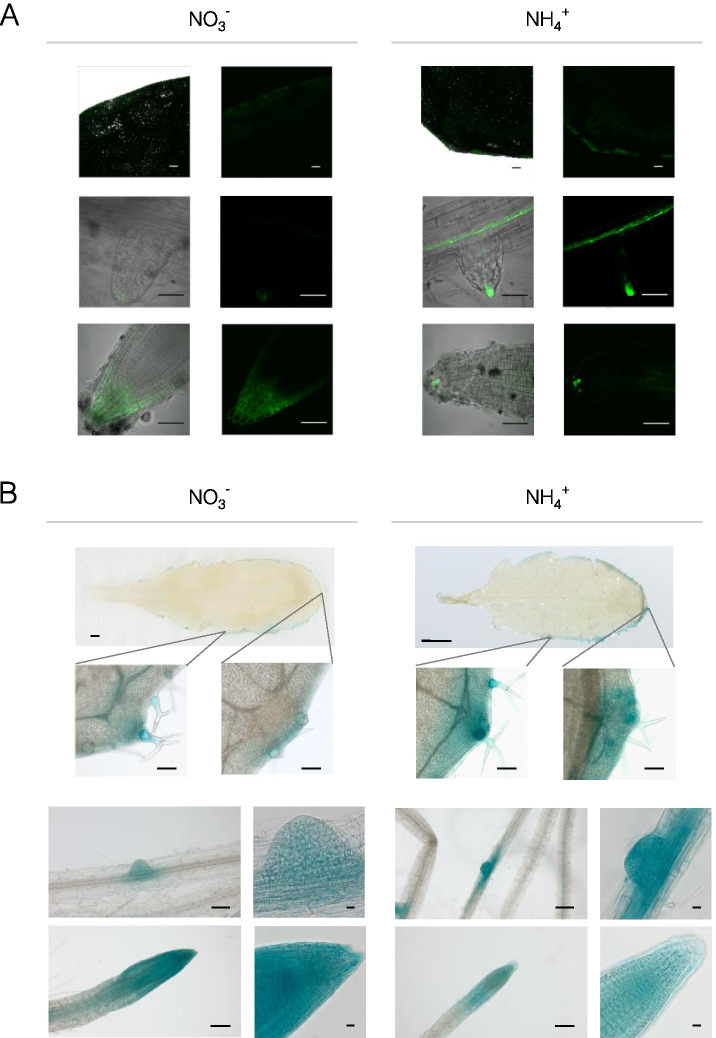
Tracing auxin-responsive reporters in tissues of transgenic *Arabidopsis thaliana* lines grown on NO_3_^−^ (control) or NH_4_^+^ as the only nitrogen source. **A** Confocal images of DR5::GFP expression. Overlay of transmission light and green fluorescence and detached green channel on the right respectively. Scale bars for leaves represent 100 μm and for roots 50 μm. **B** Photographs of DR5::GUS staining in tissues. For whole-leaf pictures scale bars represent 1 mm and for higher magnification 100 μm. Photos for root tips and lateral roots (from the differentiation zone) with developing higher-order lateral root primordia were selected. In roots scale bars for lower magnification represent 100 μm, for higher magnification 10 μm. Images shown are representative of at least five independent replicates shown in the Supplement (Supplemental Figs. 1, 2 and 3)

To examine the accumulation of auxins within tissues, Arabidopsis lines expressing *DR5::GUS* or *DR5::GFP* reporter constructs were used [27]. The auxin-mediated expression of the reporters was marginally higher in the leaves of NH_4_^+^-grown plants than in the leaves of the control plants (Fig. 2; Supplemental Figs. 1A and 2). The green fluorescence of GFP and the blue staining of GUS activity were mainly located in the leaf margins. In roots, a different reporter-specific activity dependent on the root section was observed. The root apex of the primary root showed a lower intensity of GUS and GFP color development under NH_4_^+^ nutrition (Fig. 2; Supplemental Figs. 1B and 3A). Maximum auxin responsiveness was found in the quiescent center cells of the root tip. In a mature root system most of the root system represent by lateral roots, therefore higher-order lateral roots were selected for the analysis of the differentiation zone. The induced expression of both auxin reporters was observed at the sites of lateral root formation, showing elevated staining levels in the root primordia of NH_4_^+^ -grown plants. Furthermore, higher reporter expression extended into the inner (vasculature and pericycle) tissues of the branching lateral roots in NH_4_^+^-grown plants than in the control plants (Fig. 2, Supplemental Figs. 1C and 3B).

### Auxin biosynthesis rates were decreased in leaves and upregulated in roots in the presence of ammonium

**Fig. 3:**
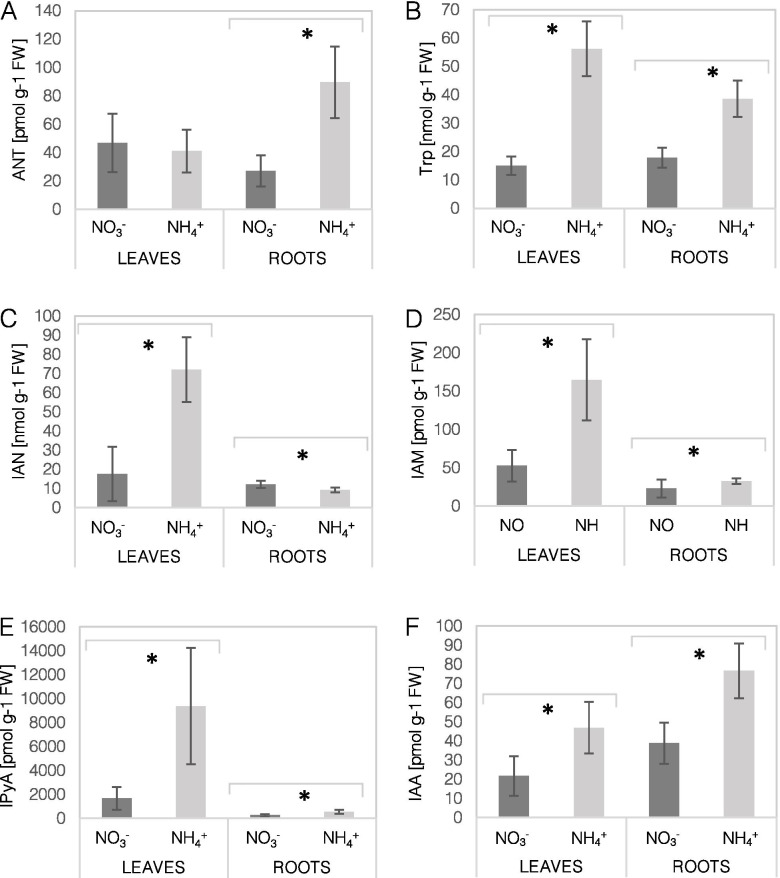
Levels of biosynthetic intermediates and free auxin in *Arabidopsis thaliana* plants cultivated on NO_3_^−^ (control) or NH_4_^+^ as the only nitrogen source. Content of anthranilate (ANT; **A**), tryptophan (Trp; **B**), indole-3-acetamide (IAM; **C**), indole-3-acetonitrile (IAN; **D**), indole-3-pyruvic acid (IPyA; **E**), and indole-3-acetic acid (IAA; **F**) in leaves and roots. Means are given with their SD (*n* = 10). Asterisks represent significant differences between NO_3_^−^ versus NH_4_^+^- derived tissues (*P* < 0.05)

We determined the levels of Trp-intermediates and free IAA in tissues of Arabidopsis plants grown with NH_4_^+^. The precursor for Trp, which is predominantly anthranilate (ANT), showed an unchanged content in leaves but was elevated in the roots of NH_4_^+^-grown plants compared to the control plants (Fig. 3A). The Trp content was almost 6-fold higher in leaves and 2-fold higher in roots under NH_4_^+^ nutrition (Fig. 3B). Further, an induced content of the derivatives indole-3-pyruvic acid (IPyA), indole-3-acetamide (IAM), and indole-3-acetonitrile (IAN) was recorded in the leaves (Fig. 3C, D, E). However, the root content of IPyA and IAM was higher, but that of IAN was lower in NH_4_^+^-grown plants than in control plants (Fig. 3C, D, E). Finally, the IAA pool was almost two times larger in the leaves and roots of NH_4_^+^-grown plants (Fig. 3F).

**Fig. 4:**
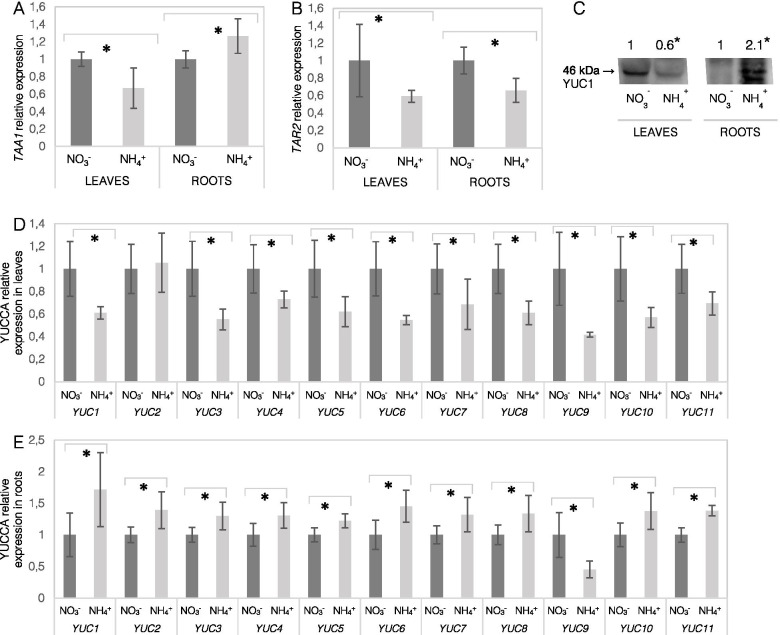
Expression of genes involved in auxin biosynthesis in the tissues of *Arabidopsis thaliana* plants grown on NO_3_^−^ (control) or NH_4_^+^ as the only nitrogen source. Transcript levels of tryptophan aminotransferase of Arabidopsis (*TAA1;* **A**) and TAA-related 2 (*TAR2;* **B**). **C** Protein level of YUC1 (46 kDa) in leaves and roots. The western blot shown is representative of three independent replicates. Transcript levels of YUCCA family of flavin monooxygenases (*YUC1-11;* **D**, **E**) in leaves and roots. Transcript abundance values are normalized to the means of the reference gene *PP2A*. Relative expression was set to 1 in control plants for reference. Means are given with their SD (*n* = 6). Asterisks represent significant differences between NO_3_^−^ versus NH_4_^+^-derived tissues (*P* < 0.05)

Subsequently, the corresponding enzymes responsible for IAA synthesis were analyzed in plant tissues. The transcript level of *TAA1* was lower in the leaves but was higher in the roots of NH_4_^+^-grown plants compared to the control plants (Fig. 4A). The level of *TAR2* transcript was lower in leaves and roots as compared to controls respectively (Fig. 4B). A similar pattern of gene expression was identified under NH_4_^+^ nutrition for almost all YUCCA genes except *YUC9* in roots. The expression of all YUCCA genes was mostly downregulated in leaves, but was higher in roots in response to NH_4_^+^ conditions (Fig. 4D and E). The same trend was observed at the protein level of a major YUC isoform, and the protein abundance of YUC1 was lower in the leaves but was higher in the roots of NH_4_^+^-grown plants (Fig. 4C).

### Ammonium induced auxin oxidation and conjugation pathways

**Fig. 5:**
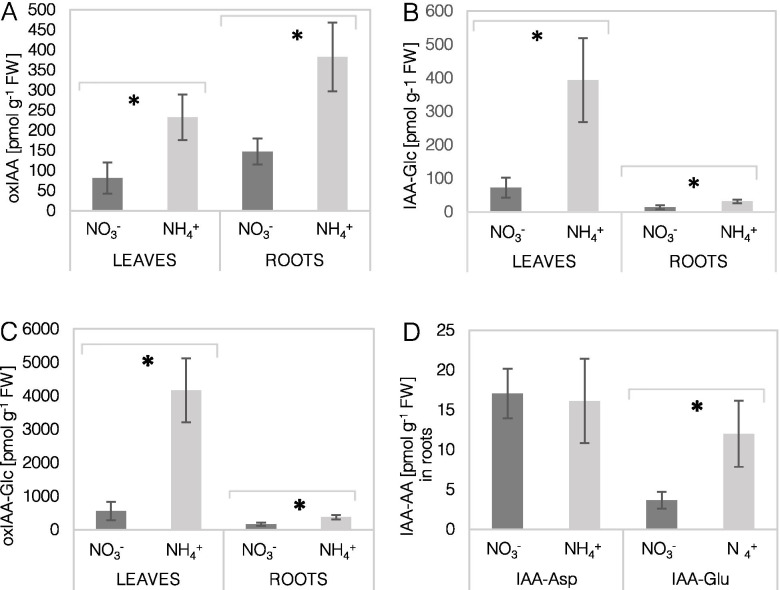
Profile of auxin metabolites in *Arabidopsis thaliana* plants grown on NO_3_^−^ (control) or NH_4_^+^ as a sole source of nitrogen. Content of oxidized indole acetic acid (oxIAA; **A**), IAA conjugate with glucose (IAA-Glc; **B**), oxidized and saccharified IAA (oxIAA-Glc; **C**), IAA conjugate with amino acids (**D**) - glutamic acid (IAA-Glu) or aspartic acid (IAA-Asp) in leaves and roots. Means are given with their SD (*n* = 10). Asterisks represent significant differences between NO_3_^−^ versus NH_4_^+^-derived tissues (*P* < 0.05)

To assess the extent to which NH_4_^+^ nutrition leads to auxin deactivation, IAA derivatives were determined in plant tissues. The oxidized pool of auxin, comprised of oxIAA, was approximately three times higher in the leaves and roots of NH_4_^+^-grown plants than in the control plants (Fig. 5A). In addition, the level of IAA-Glc was induced under NH_4_^+^ nutrition, showing a 5-fold higher content in leaves and a 2-fold higher level in roots (Fig. 5B). To a similar extent, the content of oxIAA-Glc was almost 7-fold higher in leaves and more than 2-fold higher in roots under NH_4_^+^ nutrition (Fig. 5C). Among all IAA metabolites, oxIAA-Glc and IAA-Glc showed major peaks in leaves. The content values of the conjugates IAA-Glu and IAA-Asp in roots were higher and unchanged, respectively (Fig. 5D). However, the foliar amounts of IAA-Glu and IAA-Asp were below the detection range.

**Fig. 6:**
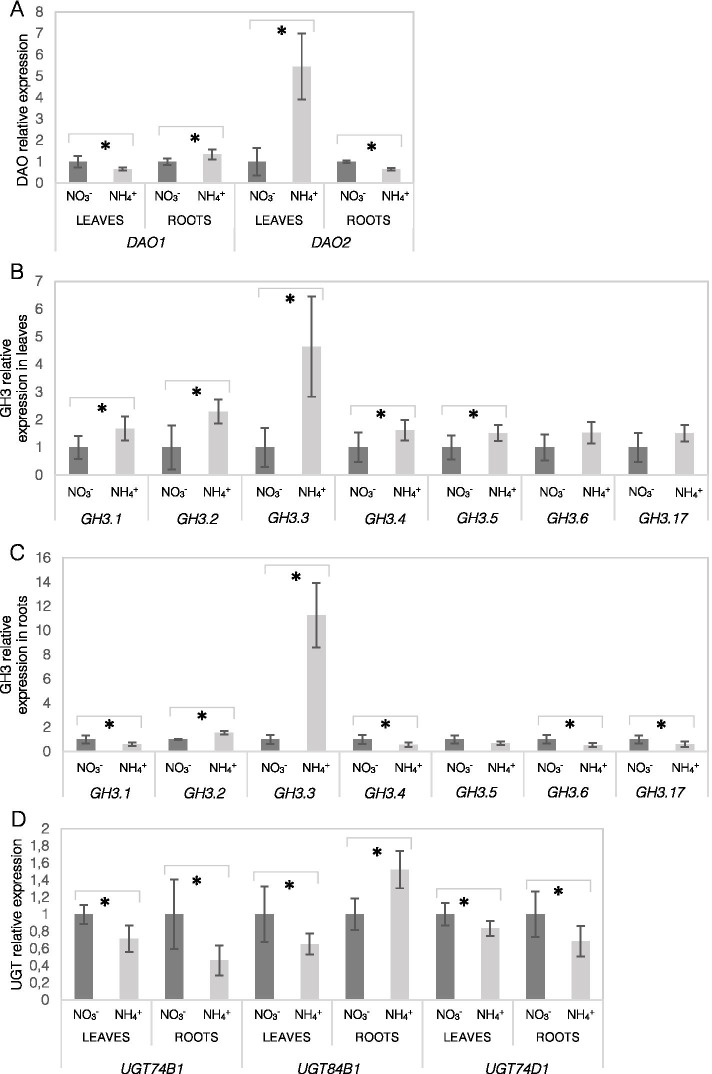
Transcriptional responses involved in auxin oxidation and conjugation in tissues of *Arabidopsis thaliana* plants grown on NO_3_^−^ (control) or NH_4_^+^ as the only nitrogen source. Relative transcript levels of dioxygenase of auxin oxidation (*DAO1-2*; **A**); Gretchen Hagen 3 (*GH3.1-6, 17*) in leaves (**B**) and roots (**C**); UDP-glucose transferases (*UTG74B1*, *UGT84B1*, and *UGT74D1*; **D**). Transcript abundance values are normalized to the means of reference gene *PP2A*. Relative expression was set to 1 in control plants for reference. Means are given with their SD (*n* = 6). Asterisks represent significant differences between NO_3_^−^ versus NH_4_^+^-derived tissues (*P* < 0.05)

After defining the enrichment of auxin catabolites in response to NH_4_^+^ nutrition, we analyzed the enzymes involved in IAA oxidative degradation and conjugation. The transcript level of *DAO1* was downregulated in leaves but was induced in roots in response to NH_4_^+^ nutrition, compared to the control plants (Fig. 6A). In contrast, *DAO2* showed more than 5-fold higher expression in leaves and was lower in roots under NH_4_^+^ conditions (Fig. 6A). The transcript levels of most GH3 were up-regulated in leaves of NH_4_^+^-grown plants (Fig. 6B). In roots *GH3.2* and *GH3.3* were strongly elevated while the expression of *GH3.1*, *4*, *6*, *17* was slightly lower and *GH3.5* showed no significant differences (Fig. 6C). The transcript levels of *UGT84B1* were lower in leaves but higher in roots in response to NH_4_^+^ nutrition (Fig. 6D). However, the transcript levels of *UGT74B1* and *UGT84D1* were decreased in both leaves and roots (Fig. 6D).

## Discussion

### Foliar auxin pool is dedicated for oxidation, storage, or root support under ammonium nutrition

IAA is a major growth regulator of plant development; therefore, it is not surprising that IAA metabolism may affect plant anatomy in response to different stress conditions [28, 29]. The decrease in the total biomass and rosette diameter of NH_4_^+^-grown plants (Fig. 1), as well as the leaf area and cell size [30], indicated the negative effects of NH_4_^+^ assimilation on plant development. It is known that auxins play an important role in inducing leaf outgrowth through the initiation of leaf primordia and may further regulate leaf shape, vein development, and other aspects of leaf expansion [31]. We observed that IAA accumulation in NH_4_^+^-grown Arabidopsis was only slightly higher (Fig. 3F), while its deposition was observed mainly in the leaf margins, which is a typical IAA pattern in young and mature leaves [31]. Further research is needed to understand the effects of IAA on leaf anatomy under NH_4_^+^ nutrition. The existing literature dealing with the NH_4_^+^ toxicity syndrome does not provide much insight into the general auxin metabolism in shoots, especially in mature plants.

Local IAA biosynthesis may participate in the regulation of plant organ development [32–34]. To understand the pattern of auxin allocation in the tissues of NH_4_^+^-grown plants, we first analyzed the events leading to IAA production. First, the higher ANT and Trp content (Fig. 3A, B) may not limit IAA biosynthesis in leaves. Overall, by investigating the expression levels of TAA-YUCCA, a lower capacity for IAA biosynthesis might be expected during NH_4_^+^ nutrition (Fig. 4). Nevertheless, all analyzed Trp-intermediates (IAN, IAM, IPyA; Fig. 3C, D, E) showed a higher content in the leaves of NH_4_^+^-grown Arabidopsis. Also the content of the final product, IAA was elevated in leaves during NH_4_^+^ nutrition (Fig. 3F). Similarly, slightly higher IAA content was found in the shoots of maize [35] and unchanged in young Arabidopsis seedlings [36]. All this indicates high synthetic rates despite lower expression of IAA synthesizing enzymes in leaves (Fig. 4). It has been shown that the expression of TAA-YUCCA is controlled through a negative feedback mechanism when IAA levels are elevated in Arabidopsis seedlings [37], which could also lower their transcript levels under NH_4_^+^ nutrition. It is important to emphasize that additional routes of the biosynthesis of IAA and other endogenous auxins may function under these specific conditions [16].

Obviously, not only the biosynthesis, but the balance between the production and degradation of auxins is responsible for regulating the endogenous IAA levels in plant tissues [16]. An interesting trend was observed for all major IAA catabolizing enzymes being downregulated in response to NH_4_^+^ nutrition (Fig. 6). The possibly lower activity of these enzymes in the leaves of NH_4_^+^-grown plants may allow IAA to accumulate, despite its lower synthesis rates. In genetic manipulation studies, these genes could severely affect the IAA accumulation in tissues [38, 39]. However, despite the low expression of DAO1, UGT84B1, UGT74D1 (Fig. 6A, D) its IAA conjugates with Glc and oxIAA-Glc and oxIAA still showed a higher pool in the leaves of NH_4_^+^-grown plants (Fig. 5A, B, C). Normally conjugated forms have a low content in tissues, but IAA increase may provoke conjugation [40]. It is not possible to measure actual IAA turnover rates, but the metabolic outcome in any case is the accumulation of a certain IAA form, which may be an indication in this study. To serve as active developmental signals, the concentration of auxins has to be tightly regulated [41].

Another way to regulate the shoot IAA content is through its export to other tissues. On the basis of lower TAA-YUCCA expression and active IAA catabolism we rather think that IAA accumulation in leaves under NH_4_^+^ nutrition may be related to changes in its transport. Usually, the size of the shoot auxin pool does not increase in the long run, but excess IAA is transported to the roots. A mechanism for IAA export to roots was recently proposed by Meier et al. [23], but the long-distance transportation role of IAA under these conditions requires further research.

### Root-derived auxin biosynthesis is directed toward its oxidation under ammonium nutrition

Events of IAA synthesis and transport occur synergistically, but under specific stress conditions, local IAA synthesis is essential for root morphology regulation [31]. Roots, similar as in leaves, provide unlimited precursor availability for IAA synthesis (Fig. 3). However, here the general pattern of the major TAA1-YUCCA genes was significantly induced (Fig. 4C, E). Similar results for TAA1, TAR2 and YUCCA enzymes were found in roots of young Arabidopsis seedlings treated with NH_4_^+^ [22]. As a result of biosynthetic reactions, the IAA content in the roots of NH_4_^+^-grown plants was strongly elevated (Fig. 3F). In contrast, in two in vitro studies the IAA contents was found to be lower in roots of NH_4_^+^-grown plants [22, 42], these differences may be related to the growth conditions or the plant developmental stage. We would rather look at auxin pools as a transient IAA state, since IAA is quickly processed. The high IAA pool could be catabolized by induced expression of DAO1, UGT84B1, GH3.2 and GH3.3 in the roots of NH_4_^+^-grown plants (Fig. 6A, C, D). An up-regulation of more GH3 and UGT isoforms was also detected in NH_4_^+^-treated Arabidopsis seedlings [22]. In a related paper it was shown that the transcription factor, WRKY46, is involved in NH_4_^+^ stress tolerance via inhibiting IAA-conjugating genes [42]. Also in rice a higher GH3 and DAO expression was associated with worse tolerance for NH_4_^+^ in sensitive cultivars [43]. As a result of conjugating reactions, IAA-Glc and oxIAA-Glc, IAA-Glu showed a higher content (Fig. 5B, C, D). The strong overproduction and irreversible degradation of IAA during NH_4_^+^ nutrition seems to be an energy-wasteful process; however, it may be thought of as a useful process if IAA is considered a signaling molecule that does not have a high shelf-life but actively regulates plant performance.

Additionally, the local IAA content in the root apical meristem, which is the site of direct IAA synthesis or a transport sink, is critical for regulation of primary root elongation [44, 45]. Auxin-metabolizing enzymes are encoded by several gene copies and are finely regulated in tissues; among the IAA-synthesizing enzymes, YUC9 is the most abundant enzyme in the root apical zone [46]. The lower YUC9 expression in the roots of NH_4_^+^-grown plants (Fig. 4E) may be indicative of decreased IAA biosynthesis in the root tip. Another indication may be expression of DAO2, which is expressed only in the root apical meristem [38, 47]. However, because of a slightly lower transcript abundance of DAO2 during NH_4_^+^ nutrition (Fig. 6A), the catabolism of auxin might not be directed toward permanent degradation. Auxin inactivation might also be expected through other IAA-metabolizing enzymes. In fact, lower IAA levels in the root tip under NH_4_^+^ nutrition can be visualized with the application of GUS or GFP sensor lines (Fig. 2). The root tips of pronounced secondary lateral roots showed a similar behavior as the primary root (results not shown). A similar pattern of lower IAA staining in the root tips has been frequently detected by GUS or GFP staining in young Arabidopsis seedlings [22–26].

On the other hand, in young lateral roots (high-order branches, results not shown) or root primordia the observed staining of sensor lines indicates higher IAA levels (Fig. 2). On this basis it can be expected that IAA acts as a signal for lateral root outgrowth in mature plants (as seen in the root phenotype in Fig. 1; Supplemental Fig. 4) in a manner alike to what was reported in seedlings under NH_4_^+^ nutrition [23]. Similar to our study, diverse agar-plate experiments have shown that NH_4_^+^ triggers an increase in lateral root numbers and inhibits primary root elongation [25, 48]. This is of general interest since higher-order lateral roots are mainly responsible for increasing root system abundance [45, 49, 50]. Therefore, a higher root branching density may compensate for the short primary root length during NH_4_^+^ nutrition and may be an adaptive response to increase root surface area. In adult Arabidopsis plants, lateral roots dominate, accounting for most of the water and nutrient uptake [51]. Therefore, plant developmental programs modulated by IAA-induced root foraging in the upper soil layer where micronutrient content is higher may be desirable [52]. Possibly stressed plants may be greedy not only for NH_4_^+^ but even more likely to take up NO_3_^−^. Thus, it might be speculated that the highly branched and dwarf NH_4_^+^-specific root design is optimal for efficient nitrogen acquisition [53, 54].

## Conclusions

In this study, we provide evidence that IAA metabolism in the tissues of mature Arabidopsis plants is affected by long-term NH_4_^+^ nutrition. The steady-state levels of free auxin in leaves and roots are controlled through a balance between anabolic and catabolic reactions. As a result, IAA overproduction simultaneously leads to the formation of IAA oxidation products, which have an even greater content. Nevertheless, transient auxin gradients including IAA maxima in root primordia and depletion in the root tip might be a signal for modulating plant anatomy in response to NH_4_^+^ stress conditions. In particular, the promotion of IAA-induced development of short root systems with highly branched lateral roots under NH_4_^+^ nutrition may be a stress-adaptive response to optimize root foraging for resources. Healthy and robust roots are key for nutrient uptake and control plant performance and growth.

## Materials and methods

### Plant material and growth conditions

*Arabidopsis thaliana* L. ecotype Col-0 (WT) plants were hydroponically cultured using an Araponics system (Araponics, Liege, Belgium). Seeds were planted on half-strength Murashige and Skoog [55] basal medium (Sigma, Darmstadt, Germany) with 1% agar and allowed to germinate in distilled water for 1 week. Thereafter, the plants were cultivated for 8 weeks in liquid medium containing 1.5 mM KH_2_PO_4_, 2.5 mM KCl, 0.7 mM CaSO_4_ · 2H_2_O, 0.8 mM MgSO_4_ · 7H_2_O, 0.06 mM NaFeEDTA, 5 mM CaCO_3_, microelement mix, and 2.5 mM Ca(NO_3_)_2_ · 4H_2_O or 2.5 mM (NH_4_)_2_SO_4_ as the nitrogen source. The growth medium was exchanged twice a week and the buffer was checked to be stable at 6.5-7 during that time. Only the application of selective media during a long-term growth regime reveals developmental differences between plants. Growth conditions were as described in our previous study [56]: a light/dark photoperiod of 8/16 h, day/night temperatures of 21/18 °C, humidity of approximately 70%, and a light intensity of 150 μmol m^− 2^ s^− 1^ photosynthetically active radiation (PAR; daylight and warm white 1:1, LF-40 W, Piła, Poland).

Plant materials, including young and mature leaves and full root systems, were collected at 12:00 pm. Leaves were directly shock-frozen in liquid nitrogen and roots were washed with water before harvest. The collected tissues were ground to powder using a mortar and pestle and stored at − 80 °C. The control group consisted of plants cultivated on NO_3_^−^, while the experimental group consisted of NH_4_^+^-grown plants (Fig. 1A).

### Visualization of auxin reporters

The Arabidopsis lines expressing the auxin-responsive *DR5::GUS* and *DR5::GFP* reporter constructs were obtained by Ottenschläger et al. [27]. Plants were grown in hydroponic culture with 5 mM NO_3_^−^ or 5 mM NH_4_^+^ as the sole nitrogen source, as described for WT plants. For staining, the leaves of the same age (same timing of appearance) and whole-root systems were used. The GUS staining procedure was performed as described by Barabasz et al. [57]. Briefly, leaves and roots were immersed in cold 90% acetone at room temperature (RT; approx. 20 °C) for 20 min, after which the samples were rinsed with 50 mM phosphate buffer (pH 7.0) containing 0.2% Triton X-100. Subsequently, tissues were infiltrated for 15 min with the GUS reaction buffer comprising 50 mM phosphate buffer (pH 7.0), 0.2% Triton X-100, and 2 mM 5-bromo-4-chloro-3-indolyl-beta-D-glucuronic acid (X-Gluc; Thermo Fisher Scientific Inc., Waltham, MA, *USA*) and incubated for 2.5 h at 37 °C. Thereafter, the tissues were cleared with decreasing ethanol concentrations and observed under a binocular microscope (Stemi 508; Zeiss, Jena, Germany).

For visualization of the *DR5::GFP* construct, GFP was excited at 488 nm and detected at 500–530 nm with a NIKON A1R MP confocal laser scanning system (Nikon, Tokyo, Japan). All adjustments of binocular- or fluorescence microscope-acquired images were performed using the software program Nis-Elements 3.22 imaging software (Nikon), with the same settings for each experimental dataset.

### Gene expression profiling

Gene expressions were measured using the real-time quantitative PCR (RT-qPCR) method. Briefly, total RNA was extracted from 100 mg of leaf and root tissues using the Plant RNA Mini Kit (Syngen, Wrocław, Poland), and 1 μg of each RNA was reverse transcribed with oligo(dT) primers using the Revert Aid H Minus First Strand cDNA Synthesis Kit (Thermo Fisher Scientific Inc.), and RNA digestion was performed using RNAse H (Sigma) as described by Escobar et al. [58] (2 U RNAse H per reaction with incubation at 37 °C for 29 min). The RT-qPCR reactions were run in the iTaq Universal SYBR Green Supermix (Bio-Rad, Hercules, CA, USA) at an amplification temperature of 60 °C. Expression levels (ΔΔCq) were analyzed using a CFX Connect Real-Time PCR System (Bio-Rad). The expression levels of the target genes were normalized with the reference gene, protein phosphatase 2A (*PP2A*, AT1G13320; [59]). The transcript abundance of each gene was expressed in relation to its corresponding abundance in the control plants (set as 1). New primers were designed for *YUC1*, *YUC2*, *YUC3*, *YUC4*, *YUC5*, *YUC6*, *YUC7*, *YUC8*, *YUC9*, *YUC10*, *YUC11*, *TAA1*, *TAR2*, *GH3.1*, *GH3.2*, *GH3.3*, *GH3.4, GH3.5, GH3.6, GH3.17, DAO1*, *DAO2*, *UGT84B1*, *UGT74B1*, and *UGT74D1*. Primer sequences and Arabidopsis accessions are listed in Supplementary Table S1.

### Auxin metabolites determination

Quantification of auxin metabolites was performed according to the method described by Novák et al. [60]. Approximately 10 mg of root or shoot tissue were homogenized and extracted with 1 mL of cold 50 mM sodium-phosphate buffer (pH 7.0) containing 0.1% sodium diethyldithiocarbamate and mixture of internal standards containing 5 pmol of [^2^H_4_]ANT, [^2^H_5_]IAM, [^2^H_4_]IPyA, [^13^C_6_]IAA, [^13^C_6_]oxIAA, [^13^C_6_]IAA-Asp, [^13^C_6_]IAA-Glu, [^13^C_6_]IAA-Glc, [^13^C_6_]oxIAA-Glc and 25 pmol of [^2^H_5_]Trp and [^2^H_4_] IAN. After centrifugation at 36000 g for 10 min, one-half of each sample was acidified with 1 M HCl to pH 2.7 and purified by solid-phase extraction (SPE) using the Oasis™ HLB columns (30 mg, 1 mL; Waters, Milford, MS, USA). For quantification of IPyA, the second half of the sample was derivatized with cysteamine (0.25 M, pH 8.0) for 1 h, acidified with 3 M HCl to pH 2.7, and purified by SPE. After evaporation under reduced pressure, the auxin content of the samples was analyzed using the 1260 Infinity II HPLC system (Agilent Technologies, CA, USA) equipped with a Kinetex C18 (50 mm × 2.1 mm, 1.7 μm; Phenomenex). The LC system was linked to a 6495 Triple Quad Detector (Agilent Technologies, USA).

### Determination of protein abundance

For western blot analysis, 200 mg of tissues were homogenized in 400 μL of 0.1 M Tris-HCl, pH 6.8. Ten microliters of each derived extract was used for electrophoresis. Proteins were separated by 10% SDS-PAGE and transferred to a polyvinylidene difluoride membrane. The membrane was blocked and probed with polyclonal rabbit antibodies raised against YUC1 (PhytoAB Inc., San Francisco, CA, USA) at a dilution of 1:1000. Goat anti-rabbit poly horseradish peroxidase secondary antibody (Bio-Rad; diluted at 1:10000) was used for chemiluminescent detection with Clarity Western ECL Substrate (Bio-Rad). Protein bands of 46 kDa were selected after background correction for a densitometric analysis, using the Image-Lab 5.2 software (Bio-Rad). The protein level of YUC1 was expressed in relation to its abundance in the control plants (set as 1).

### Statistical analysis

The significance of the differences between NO_3_^−^- and NH_4_^+^-grown plants was analyzed in both leaves and roots. The statistical analysis of data was performed using the Student’s t-test in MS Excel (Microsoft Corp., Redmond, WA, USA). The data are presented as the means and standard deviations (SD) from at least three biological repeats (indicated in each figure).

## Supplementary Information

**Additional file 1: Supplementary Table S1.** Primer sequences utilized in real-time qPCR. **Supplementary Figures 1-4.** Additional replicates for reporter staining and root phenotypes.

## Data Availability

The raw datasets for expression and metabolite levels obtained during the current study are available from the corresponding author on reasonable request. Additional replicates for presented images are available in the supplementary information file.

## Abbreviations

DAO: Dioxygenase
YUC: Flavin monooxygenase
Glc: Glucose
GFP: Green fluorescent protein
GH3: Gretchen Hagen 3
IAA: Indole-3-acetic acid
TAA: Trp aminotransferase of Arabidopsis
Trp: Tryptophan
UGT: UDP-glycosyltransferase
GUS: β-glucuronidase
