# Supplementary material for: Spatiotemporal auxin distribution in Arabidopsis tissues is regulated by anabolic and catabolic reactions under long-term ammonium stress
Source: BMC Plant Biol. 2021 Dec 18;21:602. doi: 10.1186/s12870-021-03385-9 (PMC8684078; doi:10.1186/s12870-021-03385-9)
Supplement: Supplementary file 1 — Additional file 1: Supplementary Table S1. Primer sequences utilized in real-time qPCR. Supplementary Figures 1-4. Additional replicates for reporter staining and root phenotypes. [file 12870_2021_3385_MOESM1_ESM.zip › Suplement_IAA_BMC2.pdf]

SUPPLEMENTARY MATERIALS

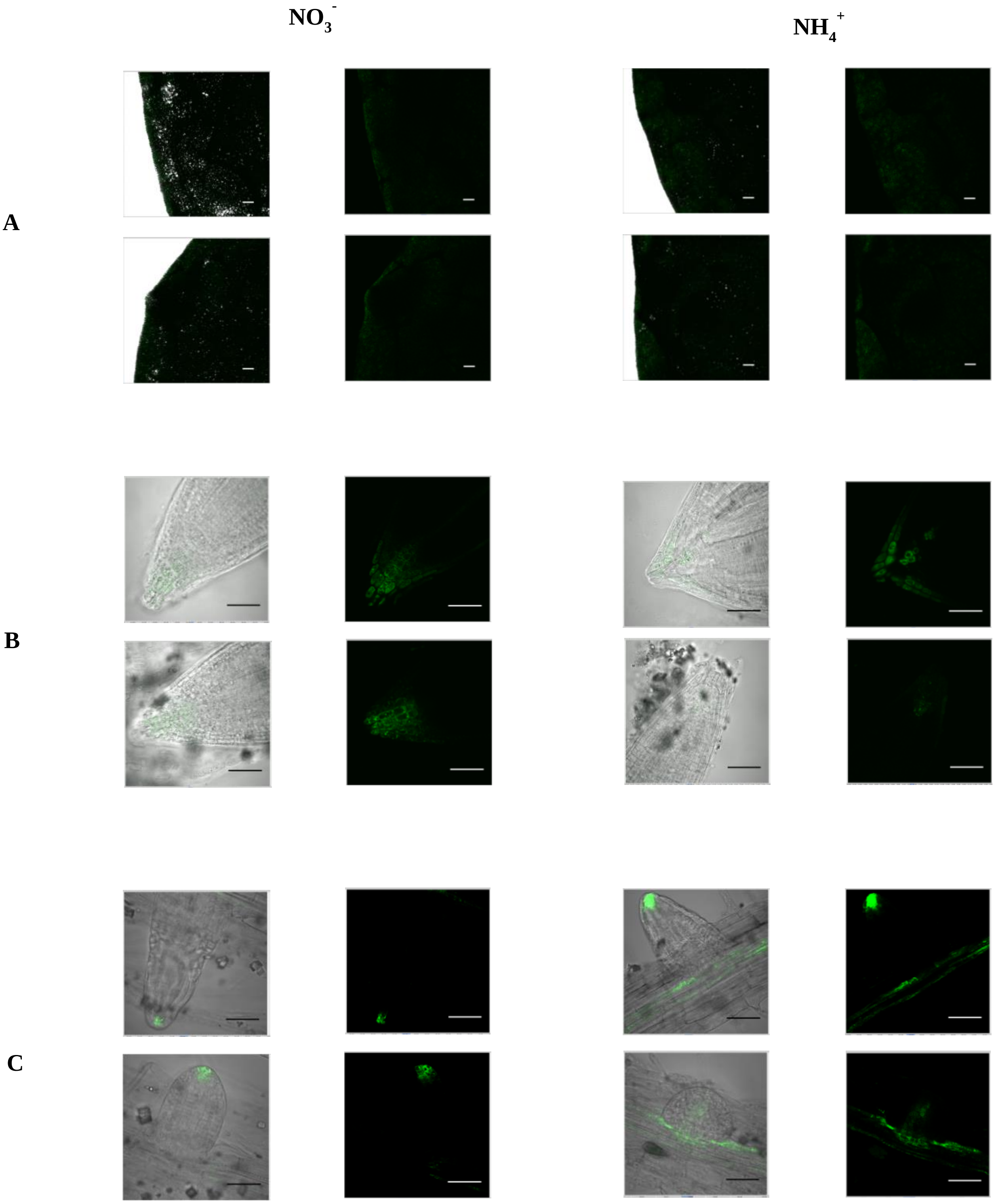

Supplementary Figure 1. Fluorescent images of DR5::GFP *A. thaliana* line representing leaves **(A)**, root tips **(B)** and lateral root primordia **(C)** of plants cultivated on NO_3^- (control) or NH_4^+ as a sole source of nitrogen. Overlay with transmission light channel and green channel on the left and green channel on the right. Scale bars for leaves represent 100 µm, for roots 50 µm.

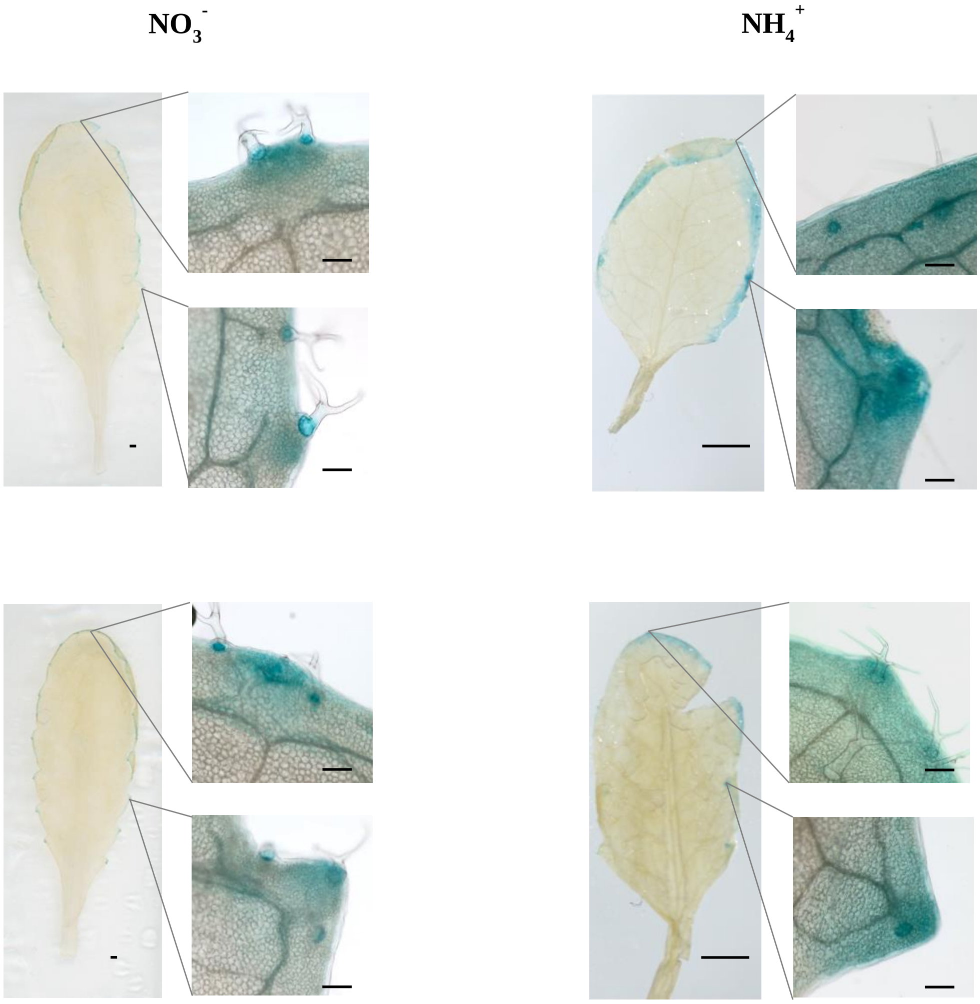

Supplementary Figure 2. Photographs of DR5::GUS staining *A. thaliana* line leaves of plants cultivated on NO_3^- (control) or NH_4^+ as a sole source of nitrogen. Scale bars for lower magnification represent 1 mm, for higher magnification 100 µm.

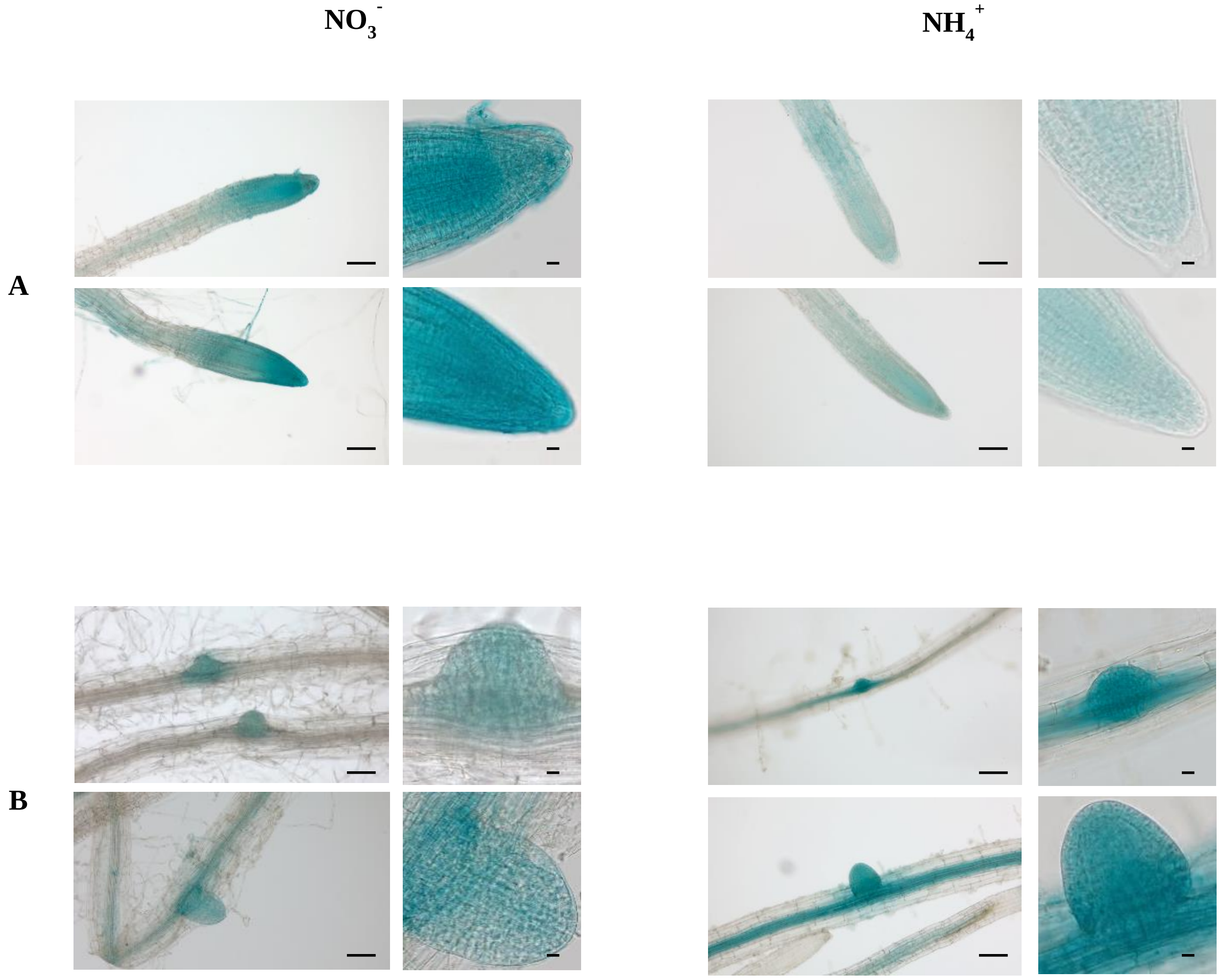

Supplementary Figure 3. Photographs of DR5::GUS staining *A. thaliana* line grown on NO_3^- (control) or NH_4^+ as a sole source of nitrogen representing root tips of primary roots **(A)** and developing higher order lateral root primordia from the differentiation zone **(B)**. Scale bars for lower magnifications represent 100 µm, for higher magnification: 10 µm.

NO_3^- **NH_4^+**

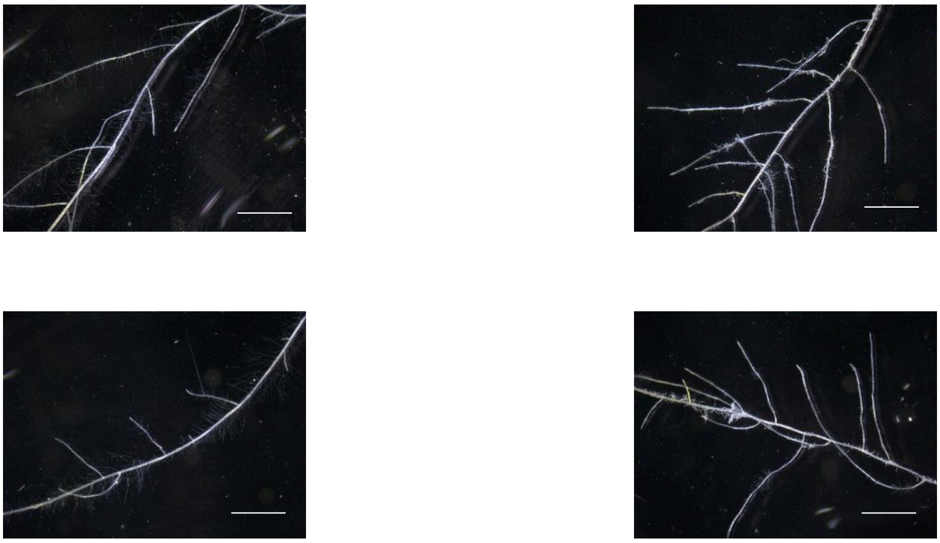

Supplementary figure 4. Additional photos of *A. thaliana* roots grown on NO_3^- (control) or NH_4^+ as a sole source of nitrogen. Scale bars represent 1 mm.

Supplemental Table S1. Primer sequences utilized in real-time qPCR.

**Gene**	**Agi**	**Left primer**	**Right primer**
TAA1	AT1G70560	5’- GTATTGGGTGGGCATTGGTG -3’	5’- TTGGGAAGAGTGAAAGCATCG -3’
TAR2	AT4G24670	5’- GTCTTTGAGCCACAACCAGCA -3’	5’- TCACAATCCACTATCCCTTCTTCA -3’
YUC1	AT4G32540	5’-GATTCAGATAATGGAAGGTGTAAAGG -3’	5’-GTCCTCTTCTCGTAAAACCAACTG -3’
YUC2	AT4G13260	5’- GTGGCTAAAGGGAGTGAACAT -3’	5’- CTCAGCGATCTTCTTGGCATC -3’
YUC3	AT1G04610	5’-GCTGTTCATGTTTTACCAAGAGAG -3’	5’-GCCTTTTTAGACCGTATTTATCG -3’
YUC4	AT5G11320	5’- GGACAAATTAAAGTGACGCAAGC -3’	5’- TGCCCCGTTCCTCGTTATT -3’
YUC5	AT5G43890	5’- GGCGAGAAATACAGAGGAAAGAG -3’	5’- TACTAGCCACAGAGGAAACCACT -3’
YUC6	AT5G25620	5’- TAAGGTGTGTTCGGGGATAAG -3’	5’- TAATGGCGTCAAATCTCTCTGTT -3’
YUC7	AT2G33230	5’- CTCCGTTCATGTATTACCGAG -3’	5’- GCTATTCCCGCTACGATTTTG -3’
YUC8	AT4G28720	5’- GGTGGCTACGGGAGAAAATG -3’	5’- GGAAGGCTTAGCGAAATGGT -3’
YUC9	AT1G04180	5’- GTTAGAAGCTCGGTTCATGTTTTAC -3’	5’-AACCAACCACGATAAGATAAGCAG-3’
YUC10	AT1G48910	5’- GCGATGGCGAGATTCAGGTT -3’	5’- AGGTCTTCCCGTTGATGCTTC -3’
YUC11	AT1G21430	5’- CGTTGTTCGTAGCCAGGTGC -3’	5’- CAGCCGACGTCAATGGTAGC -3’
DAO1	AT1G14130	5’- CAAAGGGAGATTATGGTGAAGTATG -3’	5’- TTCCTCGCTAAATCCGTTGC -3’
DAO2	AT1G14120	5’- TCGCCAGATCAAAGGGAGA -3’	5’- CAAGTCCACCAACATCCTCATC -3’
UGT84B1	AT2G23260	5’- ACGGTGGTGGCTGGTGTTC-3’	5’- TCCGCCGCTCTCCTTCTTA-3’
UGT74B1	AT1G24100	5’- TGAAGGGTTAGAAGAAACACAAGA-3’	5’- CTCAAGCCACTCCATACACTCC-3’
UGT74D1	AT2G31750	5’- CATTCCTCACCACTTCCTCCA-3’	5’- CATCGTCAATGGGGACAAAAG-3’
GH3.1	AT2G14960	5’- TGCCCGTAATGAATTTGTATG -3’	5’- CGGTGTCTTTGTTTCGGACTT -3’
GH3.2	AT4G37390	5’- TTTCTCACAAGCTCAGGAACATCT -3’	5’- GTCTTTGACTCCGACTTCACGA -3’
GH3.3	AT2G23170	5’- CATGCCTGTGATGAATCTCTACG -3’	5’- GAGCCTTGCCTTTGTCTAATCC -3’
GH3.4	AT1G59500	5’- GCTCTGGAACATCTGCTGGC-3’	5’- TTTGTCTAATCCCGGCACGTA-3’
GH3.5	AT4G27260	5’- GCTCTGGGACATCTGGTGG-3’	5’- GAACGAACTGGCTCATCACA-3’
GH3.6	AT4G27260	5’- CTGGGCTTTACAGGTACAGAGTG-3’	5’- ACCACGTTCTTGCGGCA-3’
GH3.17	AT1G28130	5’- CAAATATGTGGATGGGCTAGATG-3’	5’- ATGGTCTGGTCAGGGCTAGT-3’
