# Supplementary material for: Spatiotemporal auxin distribution in Arabidopsis tissues is regulated by anabolic and catabolic reactions under long-term ammonium stress
Source: BMC Plant Biol. 2021 Dec 18;21:602. doi: 10.1186/s12870-021-03385-9 (PMC8684078; doi:10.1186/s12870-021-03385-9)
Supplement: Supplementary file 1 — Additional file 1: Supplementary Table S1. Primer sequences utilized in real-time qPCR. Supplementary Figures 1-4. Additional replicates for reporter staining and root phenotypes. [file 12870_2021_3385_MOESM1_ESM.zip › YUC1_blot_AP3.pdf]

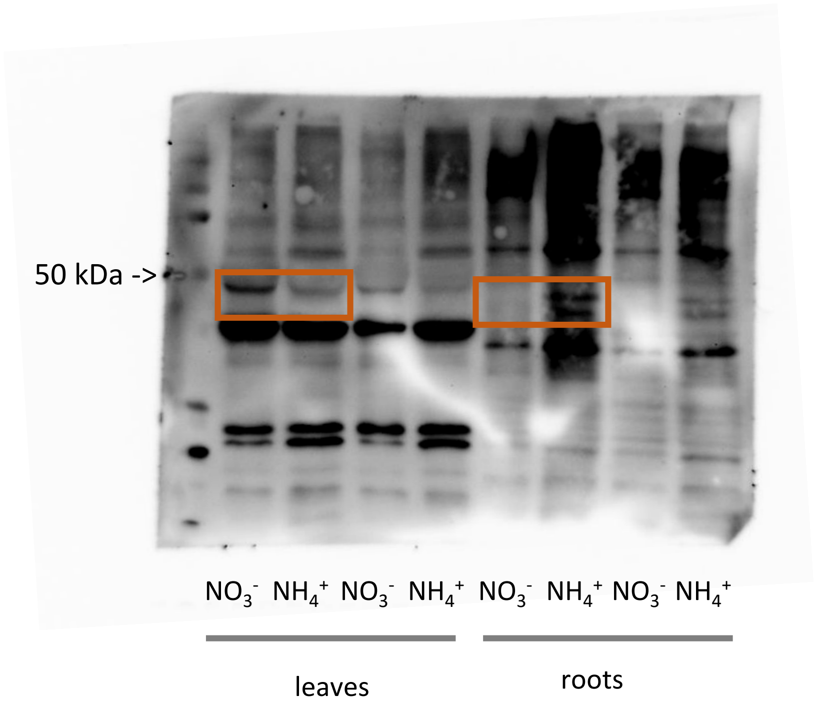

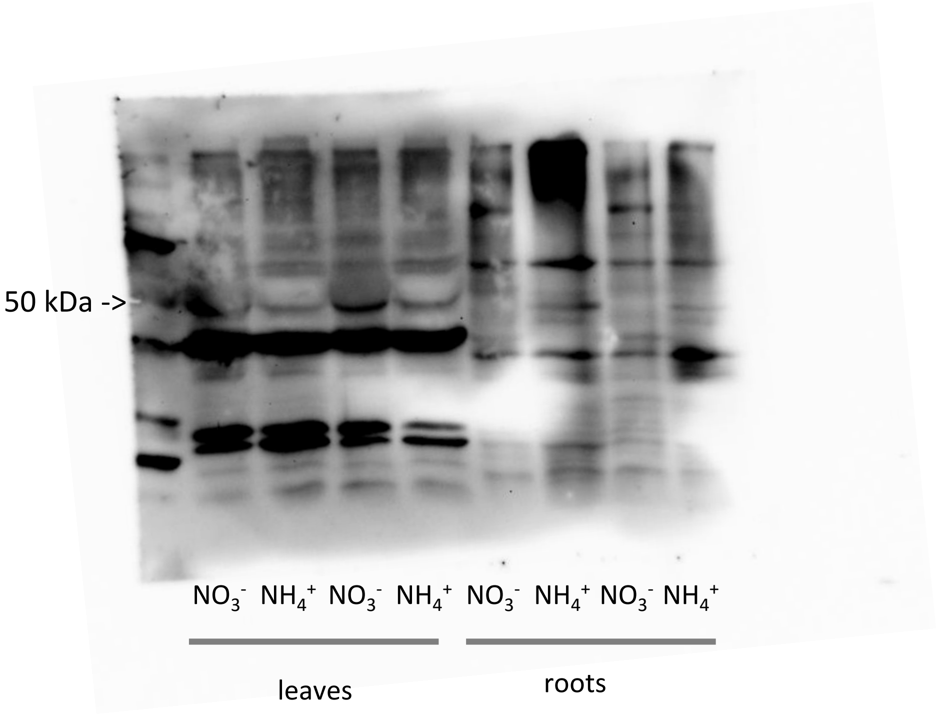

Supplementary Figure 4. Protein level of YUC1 (46 kDa) in A. thaliana grown on NO_3^- (control) or NH_4^+ as a sole source of nitrogen. All four indipendent replicates are shown for leaves and roots. Orange squares mark the bands presented the main figure 4.
